# Linguistic barriers in communication between oncologists and cancer patients with migration background in Germany: an explorative analysis based on the perspective of the oncologists from the mixed-methods study POM

**DOI:** 10.1007/s43999-022-00001-7

**Published:** 2022-06-22

**Authors:** Nicola Riccetti, Isabelle Hempler, Kerstin Hermes-Moll, Vitali Heidt, Oliver Bayer, Thomas Walawgo, Martin Merbach, Susanne Singer

**Affiliations:** 1 University Medical Centre, Institute of Medical Biostatistics, Epidemiology and Informatics (IMBEI), Mainz, Germany; 2 University Cancer Centre Mainz, Mainz, Germany; 3 Scientific Institute of Office-Based Haematologists and Oncologists (WINHO GmbH), Cologne, Germany; 4 Evangelisches Zentralinstitut Für Familienberatung, Berlin, Germany

**Keywords:** Migrants, Neoplasm, Communication barriers, Health services needs and demands, Health services accessibility

## Abstract

**Objective:**

We aimed at investigating the predictors of linguistic barriers among office-based haemato-oncologists during consultation with migrant cancer patients in Germany.

**Methods:**

Physicians from haemato-oncological practices were invited to participate in a cross-sectional study.

Linguistic barriers and family factors were ascertained using a newly developed online-questionnaire with the scales “Overall linguistic barriers”, “Self-perceived linguistic barriers” and “Family factors – antagonistic behaviour”.

Predictors of linguistic barriers were identified using multivariate ANOVA via step-wise backwards selection.

**Results:**

Fifty-five physicians participated in the study.

Treating patients from Sub-Saharan Africa predicted higher overall and self-perceived linguistic barriers (F [2,46] = 4.51, *p* = .04; and F [3,45] = 5.44, *p* = .02, respectively). Working in an single practice (F [3,45] = 4.19; *p* = .05) predicted higher self-perceived linguistic barriers. Employees who could act as translators predicted lower barriers in form of antagonistic behaviour from relatives (F [2,48] = 6.12; *p* = .02).

**Conclusions:**

The results indicate that linguistic barriers are affected by the level of linguistic concordance between patients and medical personnel. A temporary solution might be the presence of linguistically competent personnel in the practice. However, the results of this study highlight the need for greater availability of linguistic competent translators for consultations in haemato-oncological practices.

**Supplementary Information:**

The online version contains supplementary material available at 10.1007/s43999-022-00001-7.

## Introduction

A comprehensive physician–patient relationship has been found to be positively associated with patients’ treatment adherence, satisfaction with care, symptoms resolution, and mental and physical status [1–3].

However, this relationship seems to be influenced by patients’ socio-demographic characteristics, such as age, socio-economic status and ethnicity/migration background [4–8]. Specifically regarding the latter aspect, during consultations with patients from an ethnic minority (defined as a *“non-dominant, smaller group with a* <  < *shared cultural heritage, including values, traditions, and often language* >  > *” *[9]) or with migration background (defined as *“any person who is moving or has moved across an international border or within a state away from his/her habitual place of residence, regardless of (a) the person’s legal status; (b) whether the movement is voluntary or involuntary; (c) what the causes for the movement are or (d) what the length of the stay is.” *[10]), physicians were observed engaging less in building a relationship with the patient, as well as conveying fewer biomedical, informative, and supportive communication statements, compared to consultations with non-minority/non-migrant patients [6, 7, 11]. Conversely, ethnic concordance between physicians and patients has been seen to result in higher trust in the physician and a higher level of health-knowledge transferred, especially in patients with low health literacy [12]. This latter aspect becomes even more relevant when considering that health literacy, defined as the ability of a person to seek, understand, and utilize health information [13], is generally lower in patients with a migration background, compared to non-migrant patients. However, it seems to vary within different migrant groups [14–16] and can be explained to a large extent by socio-economic factors [17].

This lower health literacy, combined with the aforementioned association between the ethnicity/migration background of the patients and the relationship to the physician, might explain why patients from an ethnic minority group or with migration background report higher levels of unmet information needs, worse understanding of the information provided, lower ability to navigate the health system, and a lack of trust in medical personnel, compared to non-minority/non-migrant patients [14–16, 18–22].

However, this disparity in physician–patient relationships might not be entirely due to the patients’ ethnicity/migration background, but also to practical limitations such as communication and linguistic barriers. Oncological personnel have reported communication/linguistic barriers as the most common issue when working with migrant cancer patients [23, 24]. These barriers not only hindered the patients, but also negatively influenced the medical personnel. Oncologists and oncological nurses have reported feeling insecure in consultations with ethnic minority patients, especially as they fear to appear rude or offensive because of their lack of cultural competence [25], or unconfident about understanding patients with a migration background [26].

In addition to this, almost one oncologist in two (43%) considered cultural and/or ethnical differences as the most common communication problem with ethnic minority patients [27]. Further, family factors (e.g. relatives finding it difficult to accept a poor prognosis) were often reported as main barriers to discussing care aims [24].

As most of the studies on linguistic barriers in access to oncological care focus on the perspective of the patients, we considered it important to explore the perspective of the oncologists regarding linguistic barriers and family factors during oncological consultations with cancer patients with a migration background and their relatives.

Therefore, the aim of this analysis is to investigate predictors of linguistic barriers and the role of family factors during communication between cancer patients with a migration background and office-based haemato-oncologists.

This aim was condensed into two study questions:

1. *Which aspects are associated with linguistic barriers in communication with cancer patients with migration background and their relatives?*

2. *Which aspects are associated with the roles of the relatives in communication with cancer patients with migration background?*

## Methods

### Study design and settings

Data were collected in an anonymous, nationwide, online questionnaire as part of the mixed-methods study “Psycho-oncological support in cancer patients with migration background” (*Psychoonkologische Versorgung von Krebspatienten mit Migrationshintergund* [POM]) [28, 29].

### Population

A total of 581 haemato-oncologists in 380 practices in the networks of the Scientific Institute of Office-based Haematologists and Oncologists (*Wissenschaftliches Institut der Niedergelassenen Hämatologen und Onkologen* [WINHO]) and of the Professional Association of Office-based Haematologists and Oncologists in Germany (*Berufsverband der Niedergelassenen Hämatologen und Onkologen in Deutschland* [BNHO]) received an invitation to participate in the survey via email.

The survey took place between December 2020 and March 2021. In January 2021, all practices were reminded via email to participate.

### Ethics

The study was carried out in accordance with the Declaration of Helsinki and Good Clinical Practice guidelines. Ethical approval was obtained from the Rhineland-Palatinate State Medical Association (2019–14424).

### Online questionnaire

The online questionnaire was developed based on the results of qualitative interviews with office-based haemato-oncologists previously conducted as part of the same POM project [28, 29]. The goal of these interviews was, among others, to examine the perspective of the oncologists regarding barriers to access psycho-social support services among cancer patients with a migration background, as well as other potential supportive care needs of this specific population of patients.

The questionnaire derived from these interviews comprised the following topics: (a) socio-demographic characteristics of the physicians, (b) communication with patients with a migration background, (c) cultural differences in patients with a migration background, (d) experience with screening instruments for psychological distress, (e) the role of relatives and caregivers, and (f) experience with psycho-social support services.

The questionnaire was programmed with the software *fluxio.io* and pre-tested before being available online. No registration was needed to complete it.

### Included variables

#### Outcome of interest

Two scales were created to summarize the considered barriers: “Overall linguistic barriers” and “Overall family factors”. The first scale comprised 12 items and measured the level of perceived linguistic barriers. The second scale comprised 10 items and measured the level of perceived barriers in terms of supportive or antagonistic behaviour from the relatives during consultation.

Four subscales were created by sub-setting each scale: “Self-perceived linguistic barriers” and “Linguistic barriers perceived by the patients” from the first scale, and “Family factors – supportive behaviour” and “Family factors – antagonistic behaviour” from the second scale.

The sub-scale “Self-perceived linguistic barriers” comprised 7 items measuring the level of linguistic barriers that the physicians perceived for themselves (e.g. “Due to linguistic barriers *I* feel insecure during the consultation”). The sub-scale “Linguistic barriers perceived by the patients” comprised 4 items measuring the level of linguistic barriers that the physicians perceived on the patients’ side (e.g. “Due to linguistic barriers *the patients* feel insecure during consultation”). The subscales “Family factors – supportive behaviour” and “Family factors – antagonistic behaviour” comprised 3 and 7 items, respectively, and measured the level of perceived barriers in supportive and antagonistic behaviour from the relatives (e.g. “Relatives bring me close to the problem due to language proficiency” or “Relatives contribute to support therapy decisions”; and “Relatives dominate the conversation due to their language proficiency” or “Relatives make decisions over the will of the patients”, respectively) (Supportive information – Table A2).

All scale items were based on a 4-points Likert scale coded from 1 (most positive experience) to 4 (most negative experience); answers such as “No answer” or “Do not know” were treated as missing.

Scores for the scales and subscales were created by mean values and they were calculated only if at least half of the items were answered. All scales were recoded from 0 (“No barriers”) to 100 (“Extensive barriers”). No previous factor analysis was conducted, rather the items were selected based on their content.

#### Covariates

The following socio-demographic variables of the physicians were included in the analysis: gender, age, years of work experience (5–10 years/11–20 years/ > 20 years), physician’s place of birth (Germany/Other), and further training in psycho-oncology. In addition, the type (single practice/joint practice/medical care centres) and location (large city [> 100,000 inhabitants]/middle-large city [100,000 to 20,000 inhabitants]/small city [< 20,000 inhabitants]) of the practice they work in were included.

For the descriptive analysis, foreign languages spoken by the physicians and by the employees of the practice were coded as: “One”, “Two”, “Three or more”, and “None/Missing”. In the group comparison, the levels were summarised as “One foreign language spoken” and “Two or more foreign languages spoken”. German proficiency was ignored from the calculation.

Further, a series of multiple-choice questions were included, in which physicians were asked to report the geographical origin of the patients who they: (a) treat, (b) experienced general problems in the physician–patient relationship with, (c) had misunderstandings with, (d) felt openness towards German culture from, (e) felt respectful behaviour towards the physician from, (f) felt assertiveness from their relatives, and (g) observed higher numbers of people taking part in the consultation. For each of the aforementioned questions, physicians could choose any number of answers from a priori defined areas of origin: Germany, former-Soviet Union or former-Yugoslavia, Europe or North America, Near and Middle East (incl. Turkey and North Africa), Sub-Saharan Africa, South-East Asia, Other countries. 

The answers were recoded to include each combination of questions and answers in newly created categorical variables (e.g. “I treat patients from Germany” [yes/no/missing]).

The possibility to involve additional persons (wife/partner, husband/partner, child/children, other family members, friends, employees of the practice, physicians, and other individuals) as translators in the medical consultation was included in the analysis as single categorical variables for each of the aforementioned people (e.g. “wife/partner can translate” [always/often, rarely/never, and missing]).

The use of screening instruments to identify psycho-social support needs in patients with migration background, in opposition to other strategies (e.g. direct enquiry in consultation, signals that the patient gives during the conversation, and other methods) was included in the analysis (yes/no/missing).

### Statistical analysis

#### Reliability of the scales

Internal consistency (Cronbach’s α) was used to evaluate the reliability of the scales. A threshold of alpha (α) ≥ 0.7 was considered to represent sufficient consistency. The scale “Overall linguistic barriers” and the subscales “Self-perceived linguistic barriers” and “Family factors – antagonistic behaviour” reached an internal consistency of α ≥ 0.7 and were included in this analysis. The other scales (“Overall family factors”, “Linguistic barriers perceived by the patients”, and “Family factors – supportive behaviour”) were discarded (Table 2).

#### Sample characteristics

The sample was described in absolute and relative figures for socio-demographic characteristics of the physicians and characteristics of the practices they work in, as well as for the reported experiences with other cultures (e.g. having treated patients or having experienced misunderstandings with patients from different areas of origin), and the use of screening strategies and translators.

#### Linguistic barriers and family factors

Mean scores and standard deviations for each scale were reported for the total sample and separately by socio-demographic characteristics, characteristics of the practices, experiences with other cultures, use of screening strategies, and availability of translators.

The linguistic barriers and the family factors for the different groups of physicians, based on their socio-demographic characteristics, the characteristics of the practices, their experience with screening instruments and translators, and the area of origin of the treated patient, were tested using one-way and multivariate analysis of variance (ANOVA). As for the explorative nature of this study, the one-way ANOVA was used to purposefully pre-select potential predictors to be included in the model for the multivariate ANOVA. To avoid ignoring potentially important predictors, we considered a cut-off of *p* ≤ 0.30 rather than the classic *p* ≤ 0.20. Thus, all covariates with *p* ≤ 0.30 for each scale in the one-way ANOVA were considered as potential predictors for the multivariate model. The final model for each multivariate ANOVA was then developed in a step-wise backwards selection considering best F-test and coefficient of variation (*R*^2^) (Supplementary information – Table A4).

#### Non-participants/missing values analysis

Missing values were reported in their absolute and relative frequencies. Socio-demographic characteristic of the physicians for whom the scales were not calculated and the characteristic of the practices they work in by scale were reported (Supplementary information—Table A3). No missing value imputation was conducted.

## Results

### Sample description

Among the 60 physicians who took part in the online questionnaire, 55 (92%) answered at least 5% of the questions and were considered in this analysis.

Physicians were mostly male (65%), older than 49 years of age (73%), born in Germany (85%), and residing in cities with more than 100,000 inhabitants (65%) (Table 1).

**Table 1:** Socio-demographic characteristics of the physicians and characteristics of the practices they work in. *N* = 55

Covariates	N	%
Gender		
Female	13	23.6
Male	36	65.5
Missing	6	10.9
Age class		
30–49 years	10	18.2
50–59 years	24	43.6
60–69 years	16	29.1
Missing	5	9.1
Years of work experience		
5–10 years	4	7.3
11–20 years	10	18.2
> 20 years	37	67.3
Missing	4	7.3
Country of birth		
Germany	47	85.5
The Netherlands	1	1.8
Ukraine	1	1.8
Missing	6	10.9
Foreign languages spoken—Physicians		
One foreign language	22	40.0
Two foreign languages	5	9.1
Three or more foreign languages	3	5.4
No foreign language/Missing	25	45.5
Foreign languages spoken—Employees		
One foreign language	9	16.4
Two foreign languages	3	5.5
Three or more foreign languages	4	7.3
No foreign language/Missing	39	70.9
Further training in psycho-oncology		
No	39	70.9
Yes	11	20.0
Missing	5	9.1
Type of practice		
Single practice	5	9.1
Joint practice	38	69.1
Medical care centres	8	14.6
Missing	4	7.3
Location of practice		
Large city (> 100,000 inhabitants)	36	65.5
Middle-large city (20,000 to 100,000 inhabitants)	12	21.8
Small city (< 20,000 inhabitants)	4	7.3
Missing	3	5.5

### Experience with patients with migration background and their relatives

In the single items section, physicians reported that among their patients with a migration background, most came from the Near or Middle East (98% of physicians), the former-Soviet Union or former-Yugoslavia (96%), and Europe or North America (82%).

In addition, physicians reported having experienced problems in the patients-physician relationship most commonly with patients from the Near or Middle East (78% of physicians), Sub-Saharan Africa (33%) and the former-Soviet Union or former-Yugoslavia (24%).

**Fig. 1:**
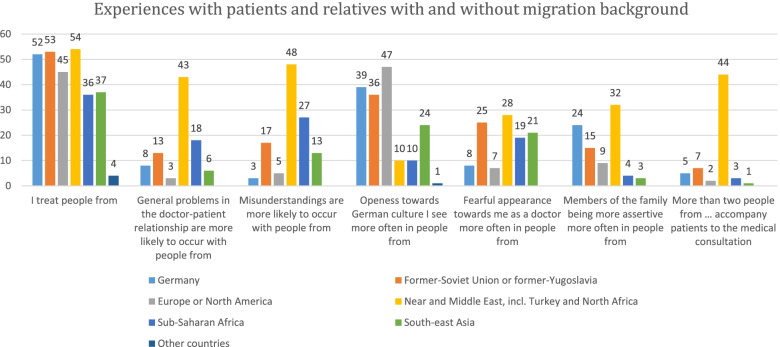
Absolute number of doctors reporting having experienced the specific situation for each of the considered group of patients and relatives with and without migration background. *N* = 55. A colourless version of this figure is included as table in the supplementary information (Table A1)

Similarly, physicians most commonly reported having experienced misunderstandings with patients from the Near or Middle East (87% of physicians), Sub-Saharan Africa (49%) and the former-Soviet Union or former-Yugoslavia (31%) (Fig. 1; Supplementary information—Table A1).

### Scales

The scale “Overall linguistic barriers” was completed by 52 physicians and had a mean value of 59.5 (Standard deviation [SD] = 22.9). The sub-scale “Self-perceived linguistic barriers” was completed by 52 physicians and had a mean value of 62.7 (SD = 19.4). The sub-scale “Family factors – antagonistic behaviour” was completed by 53 physicians and had a mean value of 50.5 (SD = 21.6) (Table 2).

**Table 2:** Psychometric characteristics and internal consistence (Cronbach’s α) of the scales. All scales range from 0 (no barriers) – 100 (extensive barriers). The scales included in the analysis are reported in bold

Name of scale	*N*	Median	Mean	Std Dev	α
**Overall linguistic barriers**	**52**	**61.7**	**59.5**	**22.9**	**0.79**
**Self-perceived linguistic barriers**	**52**	**63.8**	**62.7**	**19.4**	**0.73**
Linguistic barriers perceived by the patients	53	50.0	48.2	22.9	0.62
Overall family factors	54	43.2	42.2	21.9	0.56
Family factors – antagonistic behaviour	54	40.0	39.6	16.7	0.62
**Family factors – antagonistic behaviour**	**53**	**52.8**	**50.5**	**21.6**	**0.67**

### Barriers by socio-demographic characteristics of the physicians and characteristic of the practices

Younger physicians reported lower scores in the “Family factors – antagonistic behaviour” scale (mean scores = 39.1, 54.6 and 54.6 for physicians between 30–49, 50–59 and 60–69 years of age, respectively). Physicians working in single practices (*Einzelpraxen*) reported higher overall linguistic barriers and self-perceived linguistic barriers compared to physicians working in joint practices (*Gemeinschaftspraxen*) and medical care centres (*Medizinische Versorgungszentren [MVZ]*) (mean scores = 77.0 vs 59.6 and 62.8, and 78.3 vs 62.4 and 66.0, respectively). Physicians working in middle-large cities reported higher overall linguistic barriers (mean scores = 69.2 vs 58.8 and 56.9 compared to physicians living in large and small cities, respectively) and higher self-perceived linguistic barriers (mean scores = 73.1 vs 61.1 and 63.9 compared to physicians living in large and small cities, respectively) (Table 3).

**Table 3:** Socio-demographic characteristics of the physicians and characteristics of the practices they work in by linguistic barriers and antagonistic behaviours from the relatives. All scales coded as 0 (“no barriers/no antagonistic behaviour”) and 100 (“extensive barriers/extensive antagonistic behaviour)

Covariates	Overall linguistic barriers			Self-perceived linguistic barriers			Family factors – antagonistic behaviour		
	*N*	Mean	Std	*N*	Mean	Std	*N*	Mean	Std
Gender									
Female	12	64.4	20.6	12	68.1	18.1	13	49.6	20.3
Male	35	62.8	20.4	35	64.9	16.6	35	52.3	22.9
Missing	5	24.4	15.4	5	33.7	19.5	5	39.6	14.3
Age class									
30–49 years	10	58.9	17.1	10	65.0	16.6	10	39.1	21.1
50–59 years	23	66.3	18.1	23	67.5	13.1	24	54.6	20.6
60–69 years	15	58.7	26.6	15	61.4	23.0	15	54.6	22.8
Missing	4	24.6	17.8	4	33.4	22.5	4	38.7	16.3
Years of work experience									
5–10 years	4	61.7	9.0	4	65.7	6.9	4	36.3	24.8
11–20 years	10	66.8	19.7	10	69.8	18.4	10	50.4	23.5
> 20 years	35	60.1	22.9	35	63.0	17.9	36	53.7	20.8
Missing	3	25.1	21.7	3	30.5	26.7	3	30.8	5.4
Place of birth									
Outside Germany	*Inv*	*Inv*	*Inv*	*Inv*	*Inv*	*Inv*	*Inv*	*Inv*	*Inv*
Germany	46	62.5	21.7	46	65.0	17.8	47	51.0	21.9
Missing	5	33.6	20.3	5	41.2	24.3	5	39.6	12.7
Foreign languages spoken—Physicians									
One foreign language	21	66.5	23.9	21	67.4	18.6	22	58.6	21.3
Two foreign languages	5	59.4	23.8	5	67.0	18.6	5	58.5	14.3
Three or more foreign languages	3	53.2	9.8	3	54.2	8.3	3	27.0	6.1
No foreign language/Missing	23	53.9	22.2	23	58.5	20.9	23	44.0	20.8
Foreign languages spoken—Employees									
One foreign language	9	51.2	27.2	9	57.6	23.8	9	47.0	24.9
Two foreign languages	3	51.1	3.7	3	59.0	4.2	3	34.0	18.9
Three or more foreign languages	4	67.4	27.3	4	72.0	20.3	4	55.2	17.9
No foreign language/Missing	36	61.4	22.2	36	63.2	19.1	37	52.1	21.5
Further training in psycho-oncology									
No	38	60.6	22.5	38	63.8	17.7	38	51.4	21.6
Yes	10	63.6	20.2	10	67.0	18.6	11	55.0	21.9
Missing	4	38.3	27.7	4	40.7	28.0	4	28.8	6.0
Type of practice									
Single practice	5	77.0	16.6	5	78.3	13.5	5	56.6	8.4
Joint practice	36	59.6	20.3	36	62.4	17.7	37	50.6	22.0
Medical care centres	8	62.8	24.1	8	66.0	15.4	8	48.6	28.5
Missing	3	19.9	18.3	3	30.5	26.7	3	43.4	16.3
Location of practice									
Large city (> 100,000 inhabitants)	34	58.8	22.6	34	61.1	18.0	35	50.6	23.9
Middle-large city (20,000 to 100,000 inhabitants)	12	69.2	20.1	12	73.1	16.1	12	54.4	17.9
Small city (< 20,000 inhabitants)	4	56.9	3.2	4	63.9	5.9	4	45.8	14.2
Missing	2	18.1	25.6	2	24.7	34.9	2	34.0	0.0

*Std* Standard deviation;

Levels with < 2 physicians were not included or reported as invalid (Inv.)

Physicians treating patients from Sub-Saharan Africa reported higher overall linguistic and self-perceived linguistic barriers (mean scores = 64.8 vs 49.4 and 67.5 vs 53.5, respectively, compared to physicians reporting not treating patients from Sub-Saharan Africa) (Table 4).

**Table 4:** Country/geographical area of origin of the treated patients by linguistic barriers and antagonistic behaviours from the relatives. All scales coded as 0 (“no barriers/no antagonistic behaviour”) and 100 (“extensive barriers/extensive antagonistic behaviour). Country/geographical area of origin was assessed as reported from the physicians

Covariates	Overall linguistic barriers				Self-perceived linguistic barriers			Family factors – antagonistic behaviour		
	N	Mean		Std	N	Mean	Std	N	Mean	Std
I treat people from…										
Germany										
No	2	53.4		2.7	2	61.4	3.4	3	41.8	21.3
Yes	50	59.7		23.3	50	62.7	19.8	50	51.0	21.8
former-Soviet Union or former-Yugoslavia										
No	*Inv*	*Inv*		*Inv*	*Inv*	*Inv*	*Inv*	2	50.5	43.4
Yes	51	58.9		22.7	51	62.5	19.6	51	50.5	21.2
Europe or North America										
No	9	57.9		27.2	9	59.4	27.6	10	41.2	22.8
Yes	43	59.8		22.2	43	63.4	17.6	43	52.6	21.0
Near and Middle East (incl. Turkey and North Africa)										
No	*Inv*	*Inv*	*Inv*		*Inv*	*Inv*	*Inv*	*Inv*	*Inv*	*Inv*
Yes	52	59.5		22.9	52	62.7	19.4	52	51.1	21.4
Sub-Saharan Africa										
No	18	49.4		26.7	18	53.5	23.1	19	49.5	19.1
Yes	34	64.8		18.9	34	67.5	15.4	34	51.0	23.2
South-East Asia										
No	17	63.4		20.3	17	65.7	17.8	18	50.6	23.2
Yes	35	57.6		24.1	35	61.2	20.2	35	50.4	21.1
Other countries										
No	48	60.0		22.4	48	62.9	19.2	49	50.3	21.9
Yes	4	52.9		30.4	4	60.2	24.7	4	52.8	20.4

*Std* Standard deviation

Levels with < 2 physicians were not included or reported as invalid (Inv.)

Lower antagonistic behaviour by the relatives was reported when employees of the practice could act as translators (mean scores = 26.9 vs 53.3 for “always/often” and “rarely/never”, respectively) (Table 5).

**Table 5:** Use of screening questionnaires and presence of translators by linguistic barriers and antagonistic behaviours from the relatives. All scales coded as 0 (“no barriers/no antagonistic behaviour”) and 100 (“extensive barriers/extensive antagonistic behaviour). Availability of people acting as translators was assessed as reported from the physicians

Covariates	Overall linguistic barriers			Self-perceived linguistic barriers			Family factors – antagonistic behaviour		
	N	Mean	Std	N	Mean	Std	N	Mean	Std
Use of screening questionnaires									
Yes	7	59.9	19.7	7	63.9	17.2	7	58.2	22.4
No	45	59.4	23.5	45	62.5	19.9	45	49.9	21.3
Wife/Partner can translate									
Always/Often	23	58.0	16.6	23	62.1	12.7	23	51.1	23.5
Rarely/Never	29	60.7	27.0	29	63.1	23.7	29	51.0	20.0
Husband/Partner can translate									
Always/Often	32	56.4	20.4	32	60.5	16.9	32	51.3	22.4
Rarely/Never	20	64.5	26.1	20	66.1	22.9	20	50.7	20.2
Child/Children can translate									
Always/Often	49	60.0	23.4	49	63.2	19.8	49	51.8	21.5
Rarely/Never	3	51.1	7.4	3	54.2	8.3	3	39.6	20.4
Other family members can translate									
Always/Often	23	62.6	23.2	23	65.5	21.2	23	49.6	24.1
Rarely/Never	29	57.0	22.7	29	60.4	18.0	29	52.2	19.4
Friends can translate									
Always/Often	14	57.7	30.7	14	60.2	26.7	14	54.2	19.5
Rarely/Never	38	60.1	19.7	38	63.6	16.3	38	49.9	22.2
Employees of the practice can translate									
Always/Often	4	65.1	20.3	4	66.6	20.7	4	26.9	4.7
Rarely/Never	47	59.5	23.2	47	62.9	19.3	47	53.3	21.2
Missing	*Inv*	*Inv*	*Inv*	*Inv*	*Inv*	*Inv*	2	31.6	16.7
Physician can translate									
Always/Often	2	54.4	25.7	2	56.6	30.7	2	57.5	20.0
Rarely/Never	49	59.0	22.7	49	62.3	19.0	49	50.9	21.8
Missing	*Inv*	*Inv*	*Inv*	*Inv*	*Inv*	*Inv*	2	31.6	16.7
Other person can translate									
Always/Often	6	54.9	30.6	6	59.5	32.0	6	39.3	22.3
Rarely/Never	32	64.0	20.8	32	65.9	17.7	32	52.5	21.4
Missing	14	51.0	22.7	14	56.6	16.3	15	50.6	21.9

*Std* Standard deviation

Levels with < 2 physicians were not included or reported as invalid (Inv.)

### Analysis of variances

#### Univariate

In the one-way ANOVA, higher overall linguistic barriers and self-perceived linguistic barriers were associated with treating patients from Sub-Saharan Africa (mean scores = 64.8, 49.4; F [1, 50] = 5.88; *p* = 0.02; and mean scores = 67.5, 53.5; F [1, 50] = 6.87; *p* = 0.01, respectively).

Lower antagonistic behaviour from relatives was associated with employees acting as translators (mean scores = 26.9, 53.3; F [1, 49] = 6.06; *p* = 0.02).

No other significant association could be identified regarding the three scales and the socio-demographic characteristics of the physicians, the characteristics of the practices, the place of origin of the treated patients, and the experience with screening instruments and translations (Table 6).

**Table 6:** One-way analysis of variance (ANOVA) of socio-demographic characteristics of the physicians, characteristics of the practices they work in, experience treating patients with different origins, use of screening instruments and experiences with translation by scales. Reported are the considered levels for each covariate, the number of physicians, the mean scores, and *p*-values of the unadjusted association. Variables with *p*-values ≤ .30 were pre-selected as potential predictors to be included in the multivariate ANOVA

Covariates	Overall linguistic barriers			Self-perceived linguistic barriers			Family factors – antagonistic behaviour		
	*N*	Mean scores	*P*	*N*	Mean scores	*p*	*N*	Mean scores	*p*
Gender (female vs male)	47	64.4, 62.8	0.82	47	68.1, 64.9	0.58	48	49.6, 52.3	0.70
Age (< 50 years old vs ≥ 50 years old)	48	58.6, 64.1	0.41	48	61.4, 66.6	0.33	49	54.6, 50.0	0.51
Years of work experience (≤ 20 years vs > 20 years)	49	60.1, 65.3	0.44	49	63.0, 68.6	0.31	50	53.7, 46.4	0.29
Foreign language spoken—physicians (one vs two or more languages)	29	66.5, 57.1	0.33	29	67.4, 62.2	0.49	30	58.6, 46.7	0.18
Foreign language spoken—employees (one vs two or more languages)	16	51.2, 60.4	0.47	16	57.6, 66.4	0.42	16	47.0, 46.1	0.94
Further training in psycho-oncology (no vs yes)	48	60.6, 63.6	0.70	48	63.8, 67.0	0.62	49	51.4, 55.0	0.64
Type of practice (single practice vs other types)	49	77.0, 60.2	0.09	49	78.3, 63.1	0.06	50	56.6, 50.2	0.54
Location of the practice (large city vs medium-to-small city)	50	58.8, 66.1	0.26	50	61.1, 70.8	0.07	51	50.6, 52.2	0.80
I treat people from…									
Europe or North America (no vs yes)	52	57.9, 59.8	0.82	52	59.4, 63.3	0.58	53	41.2, 52.6	0.13
Sub-Saharan Africa (no vs yes)	52	49.4, 64.8	0.02	52	53.5, 67.5	0.01	53	49.5, 51.0	0.81
South-East Asia (no vs yes)	52	63.4, 57.6	0.39	52	65.7, 61.2	0.43	53	50.6, 50.4	0.98
Other countries (no vs yes)	52	60.0, 52.9	0.55	52	62.9, 60.2	0.80	53	50.3, 52.8	0.82
Use of screening questionnaires (no vs yes)	52	59.8, 59.4	0.96	52	63.8, 62.5	0.86	52	58.2, 49.9	0.35
Wife/partner can translate (always/often vs rarely/never)	52	58.0, 60.7	0.68	52	62.1, 63.1	0.86	52	51.1, 51.0	0.99
Husband/partner can translate (always/often vs rarely/never)	52	56.3, 64.5	0.22	52	60.5, 66.1	0.31	52	51.3, 50.7	0.92
Child/children can translate (always/often vs rarely/never)	52	60.0, 51.1	0.52	52	63.2, 54.2	0.44	52	51.7, 39.6	0.34
Other family members can translate (always/often vs rarely/never)	52	62.6, 57.0	0.38	52	65.5, 60.4	0.35	52	49.6, 52.2	0.67
Friends can translate (always/often vs rarely/never)	52	57.7, 60.1	0.73	52	60.2, 63.5	0.59	52	54.2, 49.9	0.53
Employees of the practice can translate (always/often vs rarely/never)	51	65.1, 59.5	0.64	51	66.6, 62.9	0.72	51	26.9, 53.3	0.02
Physician can translate (always/often vs rarely/never)	51	54.4, 59.0	0.78	51	56.6, 62.3	0.69	51	57.5, 50.9	0.68
Other person can translate (always/often vs rarely/never)	38	54.9, 64.0	0.36	38	59.5, 65.9	0.49	38	39.3, 52.5	0.18

#### Multivariate

The final model of the multivariate ANOVA for the scale “Overall linguistic barriers” included the type of practice the physicians were working in, and treating patients from Sub-Saharan Africa. The model was statistically significant (*p* = 0.03) and explained 14% of the variance in the sample (*R*^*2*^ = 0.14). Treating patients from Sub-Saharan Africa was a predictor of higher overall linguistic barriers (mean scores = 66.1, 53.3; F [2, 46] = 4.51; *p* = 0.04).

The final model of the multivariate ANOVA for the sub-scale “Self-perceived linguistic barriers” included the type and location of the practice, and treating patients from Sub-Saharan Africa. The model was statistically significant (*p* = 0.01) and explained 23% of the variance in the sample (*R*^*2*^ = 0.23). Working in a single practice (mean scores = 78.3, 63.1; F [3, 45] = 4.19; *p* = 0.05) and treating patients from Sub-Saharan Africa (mean scores = 68.3, 57.1; F [3, 45] = 5.44; *p* = 0.02) were predictors of higher self-perceived linguistic barriers.

The final model of the multivariate ANOVA for the scale “Family factors – antagonistic behaviour” included the presence of employees who could act as translators and treating patients from Europe and North America. The model was statistically significant (*p* = 0.03) and explained 14% of the variance (*R*^*2*^ = 0.14). The presence of employees who could act as translators resulted as predictor of lower antagonistic behaviour from the relatives (mean scores = 26.9, 53.3; F [2, 48] = 6.12; *p* = 0.02) (Table 7).

**Table 7:** Multivariate analysis of variance (MANOVA) of predictors of linguistic barriers and antagonistic behaviour from the relatives. Reported are the considered levels for each predictor, their mean scores, their F-value and *p*-value. In addition, evaluation of the whole model is included (number of records considered, coefficient of variation (*R*^*2*^), F-value and *p*-value). Model selection is documented in the supplementary information

Outcome	Predictor	Mean scores	F Value	*p*	Model evaluation			
					*N*	*R*^*2*^	F Value	*p*
Overall linguistic barriers	Type of the practice *(single practice vs other types)*	77.0, 60.2	3.26	0.08	49	0.14	3.88	0.03
	I treat patients from Sub-Saharan Africa *(yes vs no)*	66.1, 53.3	4.51	0.04				
Self-perceived linguistic barriers	Type of the practice *(single practice vs other types)*	78.3, 63.1	4.19	0.05	49	0.23	4.41	0.01
	Location of the practice *(large city vs medium-to-small city)*	61.6, 70.8	3.61	0.06				
	I treat patients from Sub-Saharan Africa *(yes vs no)*	68.3, 57.1	5.44	0.02				
Family factors – antagonistic behaviour	Employees can translate *(always/often vs rarely/never)*	26.9, 53.3	6.12	0.02	51	0.14	3.81	0.03
	I treat patients from Europa or North America *(yes vs no)*	52.8, 43.6	1.50	0.23				

## Discussion

This article aimed at exploring the predictors of linguistic barriers with cancer patients with a migration background and their relatives among office-based haemato-oncologists in Germany. Physicians treating patients from Sub-Saharan Africa reported higher overall and self-perceived linguistic barriers. Linguistic concordance between physicians and patients as well as the physicians’ familiarity with specific cultural or ethnic minority groups were previously identified as beneficial for the satisfaction with the physician–patient relationship [26, 30]. Conversely, the lack of familiarity or specific cultural knowledge was seen having a detrimental effect on the security and the feeling of empowerment of the medical personnel during the consultation [25, 26]. In this scenario, the results we observed could be interpreted in relation to the size of the various ethnic and/or migrant communities in Germany. Larger communities, such as the Turkish community (13% of resident in Germany with a migration background), the Near or Middle Eastern community (15% of resident in Germany with a migration background) or the Polish community (10% of resident in Germany with a migration background [31]), could be confronted with lower linguistic barriers during consultation. This speculation is based on the possibility of a stronger presence and support from community members, who could act as translators or provide information to patients and relatives within larger communities. At the same time, larger communities might also have higher chances of cultural and/or linguistic concordance with the physicians or the employees in the practice. Finally, the size of the community could also influence the perception of the physicians. Office-based oncologists in Germany often define ethnic minority and/or migrant cancer patients through their linguistic barriers [29], hence, in our study, a patient from a large community, with no immediate linguistic or cultural barriers during the consultation, could have been wrongly classified as non-minority/non-migrant patients from the physicians. These speculations could help to explain why in our study, we observed higher reports of overall and self-perceived linguistic barriers for consultations with patients from smaller migrant communities.

Physicians whose employees could act as translators during the consultation reported lower barriers in terms of antagonistic behaviour from relatives. According to Butow et al. [32], oncologists do not spend more time with ethnic minority patients compared to non-minority patients. This means that, because of the more numerous repetitions and the overall slower pace of the conversation, ethnic minority and/or cancer patients with migration background obtain less information during the medical consultation. In addition to this, patients and relatives from several cultures and ethnic groups consider the physician as an authority figure and struggle to enquire for further information [32, 33]. These aspects might support speculations on our results for which the employees could be seen as a reliable source of information for patients and their relatives. The possibility of an additional source of linguistically sensitive information provided by the personnel within the practice could have been beneficial for the satisfaction of the ethnic minority/migrant patients and relatives, which could in turn have resulted in less antagonistic behaviour towards the physicians. This appreciation of linguistic and culturally competent medical personnel among ethnic minority relatives of cancer patients corresponds with study results by McKenzie et al. [34] on community nurses.

Another potential explanation might be the security for the physicians to be able to rely on a trusted translator at disposal. In fact, even if the ideal solution would be to work with linguistically and culturally competent, trained and trusted translators, these professionals are not always available and are often not easy to organize [35, 36], both for organizational and financial reasons [23, 37]. Therefore, consultations with migrant patients in which the relatives act as translators are more common [23, 37, 38]. However, this might result in uncomfortable situations for the physicians [29, 35], who can feel violating the intimacy of the patients, as well as lacking trust in the accuracy of the translation [29, 35–37, 39]. Hence, our results show how employees that could act as translators might result in an easier to manage and more trusted solution to overcome linguistic and cultural barriers during the consultation, without the development of mistrust between the physicians and the relatives, and therefore with a more positive experience regarding the perceived antagonistic behaviour from the relatives.

However, when interpreting this result, it should be considered that only two physicians reported that their employees could act as translators. Hence, individual factors and experiences alone might explain the difference in mean score between the two different groups.

Similarly, physicians working in single practices reported higher self-perceived linguistic barriers compared to physicians working in joint practices or medical centres. Also for this result speculations can be drawn on whether single practices might have fewer chances for linguistic and/or cultural concordance between the patients and/or the relatives and at least one individual within the personnel of the practice.

### Implications for further research

The role of the community size and its support in overcoming barriers in access to care for different ethnic and/or migrant communities in Germany would benefit from further research. In addition to this, further research should aim at disentangling the predictors of experience during the medical consultation with cancer patients with migration background and their relatives, considering the role of patients-physician linguistic and cultural concordance as well as the role of patients’ acculturation. Also, the scales developed in this study might be considered for further testing.

In the specific German scenario, further research could look into regional difference (e.g. different federal states, or former-Western compared to former-Eastern Germany).

### Implications for the clinicians

The practical implication of the results of this study is the need for more available linguistically competent, trained and trusted translators for consultation with patients with a migration background and relatives.

A linguistically competent team in the practice might represent a temporary solution, especially to prevent antagonistic relations with the relatives of the patients.

Specific cultural competence training as well as educational material could support the medical personnel in feeling more confident when dealing with ethnic minorities and/or migrant cancer patients and their relatives. Also, cooperation with local migrant networks could result in an advantage for the practices.

However, when drawing implications from this study, it is important to consider that the study design does not allow causal conclusions. Thus, the listed implications should be considered accordingly.

### Limitations

In this study, we observed that, among office-based heamato-oncologists in Germany, working in individual practices and treating patients from Sub-Saharan Africa predicted higher linguistic barriers, and that the presence of employees who could act as translators predicted lower antagonistic behaviours from the relatives of the patients.

The generalizability of the results of this study is limited due to the small sample size and the common characteristics of the study sample (e.g. same medical specialization, same network). In addition to this, the large majority of our study sample comprised physicians born in Germany. However, patients with a migration background and low German proficiency often choose physicians who also have a migration background or proficiency in other languages. Therefore, the experience of the study sample with cancer patients with a migration background in Germany could be biased due to this aspect.

The sample size was also influenced from the missing values; however, because in the recruitment phase practices were contacted instead of single physicians, no information was available on the total number of physicians aware of the study. Hence, it was not possible to conduct an analysis of non-participant physicians.

A further limitation is the impossibility to evaluate the practical relevance of the differences in the mean scores of each scale.

In addition to this, the migration background and the area of origin of the patients and their relatives were defined by the physicians. This could have introduced recall bias and/or misclassification in this study. Also, when defining the migration background of the patients and their relatives, there was no evaluation of their level of acculturation.

All included physicians reported treating patients from areas such as the former-Soviet Union or former-Yugoslavia, and the Near or Middle East. Therefore, no group comparison could have been calculated for these areas of origin of treated patients. This means that no possible association with linguistic barriers or family factors could be observed for patients from these regions. Similarly, no assessment of any cross-cultural training among the physicians was conducted.

Finally, the format of online questionnaire is prone to selection bias in the participants.

For these reasons, the results included in this paper should be considered in light of the explorative nature of the study and their interpretation should be conducted with caution and always considering the limitation of the study sample.

## Conclusions

Among German office-based haemato-oncologists, working in individual practices and treating patients from Sub-Saharan Africa were predictors for high linguistic barriers during consultation with cancer patients with a migration background. In addition, high antagonistic behaviours from the relatives of patients were associated with the absence in the practice of employees who could act as translators.

Due to its limitations, especially in terms of the small study sample and the use of newly created scales, this study should be considered in its explorative nature. Nevertheless, our results indicate that linguistic barriers are affected by the level of linguistic concordance between patients and medical personnel and – therefore—reflect the needs for more linguistically competent supportive services for cancer patients with a migration background, especially for migrant groups with smaller communities.

### Supplementary Information

**Additional file 1:** **Table A1**. Experience of physicians with patients and relatives with and without migration background. Reported are the relative and absolute numbers of the physicians of the total study sample, answering "yes" to the specific question. (*N* = 55). **Table A2.** Psychometric characteristics (number or records [N], mean value and standard deviation [SD]) of the items included in each scale and the internal consistency (raw and standardized [Std]) of the scales with and without erased variables. All scales range from 0 (no barriers) – 100 (extensive barriers). **Table A3.** Socio-demographic characteristic of the physicians who did not complete at least 50% of the items of each scale and therefore for which the scales were not calculated, and characteristic of the practices they work in by scale. **Table A4.** Stepwise backwards model selection for the multivariate analysis of variance (ANOVA). Reported in italic the intermediate model selection and in bold the selected final model. 

## Data Availability

The data that support the findings of this study are available on request from the corresponding author. The data are not publicly available due to privacy or ethical restrictions.
