# Supplementary material for: Linguistic barriers in communication between oncologists and cancer patients with migration background in Germany: an explorative analysis based on the perspective of the oncologists from the mixed-methods study POM
Source: Res Health Serv Reg. 2022 Jun 22;1:3. doi: 10.1007/s43999-022-00001-7 (PMC11264866; doi:10.1007/s43999-022-00001-7)
Supplement: Supplementary file 1 — Additional file 1: Table A1. Experience of physicians with patients and relatives with and without migration background. Reported are the relative and absolute numbers of the physicians of the total study sample, answering "yes" to the specific question. (N = 55). Table A2. Psychometric characteristics (number or records [N], mean value and standard deviation [SD]) of the items included in each scale and the internal consistency (raw and standardized [Std]) of the scales with and without erased variables. All scales range from 0 (no barriers) – 100 (extensive barriers). Table A3. Socio-demographic characteristic of the physicians who did not complete at least 50% of the items of each scale and therefore for which the scales were not calculated, and characteristic of the practices they work in by scale. Table A4. Stepwise backwards model selection for the multivariate analysis of variance (ANOVA). Reported in italic the intermediate model selection and in bold the selected final model. [file 43999_2022_1_MOESM1_ESM.docx]

# Supplementary Information (SI)

| **Table A1**. Experience of physicians with patients and relatives with and without migration background. Reported are the relative and absolute numbers of the physicians of the total study sample, answering "yes" to the specific question. (*N* = 55) | | |
| --- | --- | --- |
| Variables | *N* | % |
| I treat people from | 3 | 5.5 |
| Germany | 52 | 94.5 |
| Former-Soviet Union or former-Yugoslavia | 53 | 96.4 |
| Europe or North America | 45 | 81.8 |
| Near and Middle East (incl. Turkey and North Africa) | 54 | 98.2 |
| Sub-Saharan Africa | 36 | 65.5 |
| South-east Asia | 37 | 67.3 |
| Other countries | 4 | 7.3 |
| General problems in the physician-patient relationship are more likely to occur with people from | 47 | 85.5 |
| Germany | 8 | 14.5 |
| Former-Soviet Union or former-Yugoslavia | 13 | 23.6 |
| Europe or North America | 3 | 5.5 |
| Near and Middle East (incl. Turkey and North Africa) | 43 | 78.2 |
| Sub-Saharan Africa | 18 | 32.7 |
| South-east Asia | 6 | 10.9 |
| Misunderstandings are more likely to occur with people from | 52 | 94.5 |
| Germany | 3 | 5.5 |
| Former-Soviet Union or former-Yugoslavia | 17 | 30.9 |
| Europe or North America | 5 | 9.1 |
| Near and Middle East (incl. Turkey and North Africa) | 48 | 87.3 |
| Sub-Saharan Africa | 27 | 49.1 |
| South-east Asia | 13 | 23.6 |
| Openness towards German culture I see more often in people from | 16 | 29.1 |
| Germany | 39 | 70.9 |
| Former-Soviet Union or former-Yugoslavia | 36 | 65.5 |
| Europe or North America | 47 | 85.5 |
| Near and Middle East (incl. Turkey and North Africa) | 10 | 18.2 |
| Sub-Saharan Africa | 10 | 18.2 |
| South-east Asia | 24 | 43.6 |
| Other countries | 1 | 1.8 |
| Fearful appearance towards me as physician more often in people from | 47 | 85.5 |
| Germany | 8 | 14.5 |
| Former-Soviet Union or former-Yugoslavia | 25 | 45.5 |
| Europe or North America | 7 | 12.7 |
| Near and Middle East (incl. Turkey and North Africa) | 28 | 50.9 |
| Sub-Saharan Africa | 19 | 34.5 |
| South-east Asia | 21 | 38.2 |
| Members of the family being more assertive more often in people from | 31 | 56.4 |
| Germany | 24 | 43.6 |
| Former-Soviet Union or former-Yugoslavia | 15 | 27.3 |
| Europe or North America | 9 | 16.4 |
| Near and Middle East (incl. Turkey and North Africa) | 32 | 58.2 |
| Sub-Saharan Africa | 4 | 7.3 |
| South-east Asia | 3 | 5.5 |
| More than two people in colloquium more often in people from | 50 | 90.9 |
| Germany | 5 | 9.1 |
| Former-Soviet Union or former-Yugoslavia | 7 | 12.7 |
| Europe or North America | 2 | 3.6 |
| Near and Middle East (incl. Turkey and North Africa) | 44 | 80.0 |
| Sub-Saharan Africa | 3 | 5.5 |
| South-east Asia | 1 | 1.8 |

| **Table A2.** Psychometric characteristics (number or records [N], mean value and standard deviation [SD]) of the items included in each scale and the internal consistency (raw and standardized [Std]) of the scales with and without erased variables. All scales range from 0 (no barriers) – 100 (extensive barriers). | | | | | | | | | | |  |
| --- | --- | --- | --- | --- | --- | --- | --- | --- | --- | --- | --- |
| Scales and  subscales | Cronbach's  alpha | | Items | N | Mean | SD | Cronbach's alpha with  erased variable | | | | |
|  |  |  |  |  |  |  | Raw variable | | Std variable | | |
|  | Raw | Std |  |  |  |  | Correlation  with total | alpha | Correlation  with total | alpha | |
| Overall linguistic  barriers | 0.78 | 0.79 | Due to linguistic barriers communication with patients with migration background is complicated | 53 | 50.9 | 18.3 | 0.29 | 0.78 | 0.26 | 0.80 | |
|  |  |  | Due to linguistic barriers I need to conduct more checks | 52 | 50.0 | 24.3 | 0.19 | 0.79 | 0.15 | 0.81 | |
|  |  |  | Due to linguistic barriers it is harder to create a trust-bond with the patients | 53 | 34.9 | 30.4 | 0.41 | 0.77 | 0.44 | 0.78 | |
|  |  |  | Due to linguistic barriers I am less satisfied | 51 | 50.3 | 23.5 | 0.53 | 0.76 | 0.54 | 0.77 | |
|  |  |  | Due to linguistic barriers I am more misunderstood | 49 | 61.2 | 49.2 | 0.56 | 0.75 | 0.56 | 0.76 | |
|  |  |  | Due to linguistic barriers patients are unsatisfied | 48 | 39.6 | 49.4 | 0.51 | 0.76 | 0.51 | 0.77 | |
|  |  |  | Due to linguistic barriers patients are misunderstood | 50 | 72.0 | 45.4 | 0.58 | 0.74 | 0.55 | 0.77 | |
|  |  |  | Due to linguistic barriers treatment decisions are more complicated | 52 | 60.3 | 21.9 | 0.41 | 0.77 | 0.45 | 0.78 | |
|  |  |  | Due to linguistic barriers I do not understand fear or distress | 53 | 59.1 | 21.3 | 0.58 | 0.76 | 0.61 | 0.76 | |
|  |  |  | Due to linguistic barriers I treat patients differently | 50 | 33.3 | 19.0 | 0.31 | 0.78 | 0.32 | 0.79 | |
|  |  |  | Due to linguistic barriers I am insecure in the colloquium | 49 | 59.2 | 33.3 | 0.58 | 0.75 | 0.57 | 0.76 | |
| Linguistic barriers  perceived on the patients | 0.59 | 0.62 | Due to linguistic barriers communication with MMH is complicated | 53 | 50.9 | 18.3 | 0.30 | 0.60 | 0.31 | 0.62 | |
|  |  |  | Due to linguistic barriers it is harder to create a trust-bond with the patients | 53 | 34.9 | 30.4 | 0.42 | 0.50 | 0.44 | 0.53 | |
|  |  |  | Due to linguistic barriers patients are unsatisfied | 48 | 39.6 | 49.4 | 0.46 | 0.46 | 0.44 | 0.52 | |
|  |  |  | Due to linguistic barriers patients are misunderstood | 50 | 72.0 | 45.4 | 0.43 | 0.48 | 0.42 | 0.54 | |
| Self-perceived  linguistic barriers | 0.70 | 0.73 | Due to linguistic barriers I need to conduct more checks | 52 | 50.0 | 24.3 | 0.20 | 0.71 | 0.16 | 0.76 | |
|  |  |  | Due to linguistic barriers I am less satisfied | 51 | 50.3 | 23.5 | 0.42 | 0.67 | 0.43 | 0.70 | |
|  |  |  | Due to linguistic barriers I am more misunderstood | 49 | 61.2 | 49.2 | 0.53 | 0.66 | 0.53 | 0.67 | |
|  |  |  | Due to linguistic barriers treatment decisions are more complicated | 52 | 60.3 | 21.9 | 0.44 | 0.67 | 0.46 | 0.69 | |
|  |  |  | Due to linguistic barriers I do not understand fear or distress | 53 | 59.1 | 21.3 | 0.57 | 0.64 | 0.60 | 0.66 | |
|  |  |  | Due to linguistic barriers I treat patients differently | 50 | 33.3 | 19.0 | 0.33 | 0.69 | 0.37 | 0.71 | |
|  |  |  | Due to linguistic barriers I am insecure in the colloquium | 49 | 59.2 | 33.3 | 0.56 | 0.62 | 0.56 | 0.67 | |
| Overall family  factor | 0.54 | 0.56 | Relatives being me close to the problem due to language proficiency | 54 | 11.1 | 31.7 | 0.11 | 0.56 | 0.14 | 0.56 | |
|  |  |  | Relatives contribute to support therapy decision | 54 | 50.0 | 19.4 | 0.17 | 0.53 | 0.18 | 0.55 | |
|  |  |  | Relatives contribute to trust in the therapy | 53 | 43.4 | 19.7 | 0.13 | 0.54 | 0.16 | 0.55 | |
|  |  |  | Relatives tell me their problems | 54 | 48.8 | 21.2 | 0.19 | 0.53 | 0.19 | 0.55 | |
|  |  |  | Relatives treat me with respect | 54 | 45.4 | 24.3 | 0.19 | 0.53 | 0.18 | 0.55 | |
|  |  |  | Relatives dominate the conversation due to their language proficiency | 54 | 53.1 | 22.0 | 0.16 | 0.54 | 0.15 | 0.56 | |
|  |  |  | Relatives make decisions over the will of the patients | 53 | 50.0 | 27.7 | 0.45 | 0.45 | 0.47 | 0.48 | |
|  |  |  | I need to inform relatives about the ethical rules of the German healthcare system | 52 | 58.7 | 32.4 | 0.20 | 0.53 | 0.20 | 0.54 | |
|  |  |  | I need to inform relatives about the importance of a correct translation | 53 | 49.1 | 21.3 | 0.40 | 0.48 | 0.37 | 0.50 | |
|  |  |  | Relatives ask me not to convey negative information | 52 | 54.8 | 26.7 | 0.24 | 0.52 | 0.24 | 0.53 | |
|  |  |  | Conveying negative information is harder in a patriarchal structured family | 50 | 48.0 | 21.5 | 0.34 | 0.49 | 0.33 | 0.51 | |
| Family factor -  supporting behaviour | 0.58 | 0.63 | Relatives being me close to the problem due to language proficiency | 54 | 11.1 | 31.7 | 0.33 | 0.67 | 0.34 | 0.66 | |
|  |  |  | Relatives contribute to support therapy decision | 54 | 50.0 | 19.4 | 0.62 | 0.20 | 0.64 | 0.22 | |
|  |  |  | Relatives contribute to trust in the therapy | 53 | 43.4 | 19.7 | 0.31 | 0.58 | 0.36 | 0.63 | |
| Family factor -  antagonistic behaviour | 0.66 | 0.67 | Relatives dominate the conversation due to their language proficiency | 54 | 53.1 | 22.0 | 0.31 | 0.64 | 0.31 | 0.66 | |
|  |  |  | Relatives make decisions over the will of the patients | 53 | 50.0 | 27.7 | 0.51 | 0.57 | 0.53 | 0.58 | |
|  |  |  | I need to inform relatives about the ethical rules of the German healthcare system | 52 | 58.7 | 32.4 | 0.45 | 0.59 | 0.46 | 0.60 | |
|  |  |  | I need to inform relatives about the importance of a correct translation | 53 | 49.1 | 21.3 | 0.46 | 0.60 | 0.45 | 0.61 | |
|  |  |  | Relatives ask me not to convey negative information | 52 | 54.8 | 26.7 | 0.24 | 0.67 | 0.25 | 0.68 | |
|  |  |  | Conveying negative information is harder in a patriarchal structured family | 50 | 48.0 | 21.5 | 0.40 | 0.61 | 0.40 | 0.63 | |

| **Table A3**. Socio-demographic characteristics of the physicians who did not complete at least 50% of the items of each scale and, therefore, for which the scales were not calculated, and characteristic of the practices they work in by scale | | | | | | | | | | | | | | |
| --- | --- | --- | --- | --- | --- | --- | --- | --- | --- | --- | --- | --- | --- | --- |
| Covariates | | | Overall linguistic barriers | | | | Self-perceived linguistic barriers | | | | Family factor  antagonistic behaviour | | | |
|  |  |  | N | | % | | N | | % | | N | | % | |
| Gender | | |  | |  | |  | |  | |  | |  | |
| Female | | | 1 | | 33.3 | | 1 | | 33.3 | | 0 | | 0 | |
| Male | | | 1 | | 33.3 | | 1 | | 33.3 | | 1 | | 50.0 | |
| Missing | | | 1 | | 33.3 | | 1 | | 33.3 | | 1 | | 50.0 | |
| Age class | | |  | |  | |  | |  | |  | |  | |
| 30-49 years | | | 0 | | 0 | | 0 | | 0 | | 0 | | 0 | |
| 50-59 years | | | 1 | | 33.3 | | 1 | | 33.3 | | 0 | | 0 | |
| 60-69 years | | | 1 | | 33.3 | | 1 | | 33.3 | | 1 | | 50.0 | |
| Missing | | | 1 | | 33.3 | | 1 | | 33.3 | | 1 | | 50.0 | |
| Years of work experience | | |  | |  | |  | |  | |  | |  | |
| 5-10 years | | | 0 | | 0 | | 0 | | 0 | | 0 | | 0 | |
| 11-20 years | | | 0 | | 0 | | 0 | | 0 | | 0 | | 0 | |
| >20 years | | | 2 | | 66.7 | | 2 | | 66.7 | | 1 | | 50.0 | |
| Missing | | | 1 | | 33.3 | | 1 | | 33.3 | | 1 | | 50.0 | |
| Place of birth | | |  | |  | |  | |  | |  | |  | |
| Outside Germany | | | 1 | | 33.3 | | 1 | | 33.3 | | 1 | | 50.0 | |
| Germany | | | 1 | | 33.3 | | 1 | | 33.3 | | 0 | | 0 | |
| Missing | | | 1 | | 33.3 | | 1 | | 33.3 | | 1 | | 50.0 | |
| Foreign languages spoken - Physician | | |  | |  | |  | |  | |  | |  | |
| One foreign language | | | 1 | | 33.3 | | 1 | | 33.3 | | 0 | | 0 | |
| Two foreign languages | | | 0 | | 0 | | 0 | | 0 | | 0 | | 0 | |
| Three or more foreign languages | | | 0 | | 0 | | 0 | | 0 | | 0 | | 0 | |
| None/Missing | | | 2 | | 66.7 | | 2 | | 66.7 | | 2 | | 100 | |
| Foreign languages spoken - Employees | | |  | |  | |  | |  | |  | |  | |
| One foreign language | | | 0 | | 0 | | 0 | | 0 | | 0 | | 0 | |
| Two foreign languages | | | 0 | | 0 | | 0 | | 0 | | 0 | | 0 | |
| Three or more foreign languages | | | 0 | | 0 | | 0 | | 0 | | 0 | | 0 | |
| None/Missing | | | 3 | | 100 | | 3 | | 100 | | 2 | | 100 | |
| Further training in psycho-oncology | | |  | |  | |  | |  | |  | |  | |
| No | 1 | | 33.3 | | 1 | | 33.3 | | 1 | | 50.0 | |  |  |
| Yes | 1 | | 33.3 | | 1 | | 33.3 | | 0 | | 0 | |  |  |
| Missing | 1 | | 33.3 | | 1 | | 33.3 | | 1 | | 50.0 | |  |  |
| Type of practice | | |  | |  | |  | |  | |  | |  | |
| Single practice | | | 0 | | 0 | | 0 | | 0 | | 0 | | 0 | |
| Joint practice | | | 2 | | 66.7 | | 2 | | 66.7 | | 1 | | 50.0 | |
| Medical care centre | | | 0 | | 0.0 | | 0 | | 0.0 | | 0 | | 0.0 | |
| Missing | | | 1 | | 33.3 | | 1 | | 33.3 | | 1 | | 50.0 | |
| Location of practice | | |  | |  | |  | |  | |  | |  | |
| Large city (>100,000 inhabitants) | | | 2 | | 66.7 | | 2 | | 66.7 | | 1 | | 50.0 | |
| Middle-large city (100,000 to 20,000 inhabitants) | | | 0 | | 0 | | 0 | | 0 | | 0 | | 0 | |
| Small city (<20,000 inhabitants) | | | 0 | | 0 | | 0 | | 0 | | 0 | | 0 | |
| Missing | | | 1 | | 33.3 | | 1 | | 33.3 | | 1 | | 50.0 | |

| **Table A4**. Stepwise backwards model selection for the multivariate analysis of variance (MANOVA). Reported in italic the intermediate model selection and in bold the selected final model. | | | | | | | | | | |  |
| --- | --- | --- | --- | --- | --- | --- | --- | --- | --- | --- | --- |
| Outcome | Predictors | | | | Model evaluation | | | | |  |  |
|  |  | | | | | *N* | *R^2^* | F Value | *p* | | |
| Overall linguistic barriers | Type of practise (Individual practice vs other types) | Location of the practise (Large city vs Medium to small city) | I treat people from Sub-Saharan Africa (No vs Yes) | Husband/Partner can translate (Aways/Often vs Rarely/Never) | | 49 | 0.17 | 2.34 | 0.07 | | |
|  | *Type of practise (Individual practice vs other types)* | *Location of the practise (Large city vs Medium to small city)* | *I treat people from Sub-Saharan Africa (No vs Yes)* | *--* | | *49* | *0.16* | *2.96* | *0.04* | | |
|  | Type of practise (Individual practice vs other types) | Location of the practise (Large city vs Medium to small city) | -- | Husband/Partner can translate (Aways/Often vs Rarely/Never) | | 49 | 0.09 | 1.51 | 0.22 | | |
|  | Type of practise (Individual practice vs other types) | -- | I treat people from Sub-Saharan Africa (No vs Yes) | Husband/Partner can translate (Aways/Often vs Rarely/Never) | | 49 | 0.15 | 2.75 | 0.05 | | |
|  | -- | Location of the practise (Large city vs Medium to small city) | I treat people from Sub-Saharan Africa (No vs Yes) | Husband/Partner can translate (Aways/Often vs Rarely/Never) | | 50 | 0.11 | 1.84 | 0.15 | | |
|  | **Type of practise (Individual practice vs other types)** | **--** | **I treat people from Sub-Saharan Africa (No vs Yes)** | **--** | | **49** | **0.14** | **3.88** | **0.03** | | |
|  | Type of practise (Individual practice vs other types) | Location of the practise (Large city vs Medium to small city) | -- | -- | | 49 | 0.08 | 2.03 | 0.14 | | |
|  | -- | Location of the practise (Large city vs Medium to small city) | I treat people from Sub-Saharan Africa (No vs Yes) | -- | | 50 | 0.09 | 2.34 | 0.11 | | |
| Self-perceived linguistic barriers | **Type of practise (Individual practice vs other types)** | **Location of the practise (Large city vs Medium to small city)** | **I treat people from Sub-Saharan Africa (No vs Yes)** | **--** | | **49** | **0.23** | **4.41** | **0.01** | | |
|  | Type of practise (Individual practice vs other types) | -- | I treat people from Sub-Saharan Africa (No vs Yes) | -- | | 49 | 0.16 | 4.55 | 0.02 | | |
|  | Type of practise (Individual practice vs other types) | Location of the practise (Large city vs Medium to small city) | -- | -- | | 49 | 0.13 | 3.56 | 0.04 | | |
|  | -- | Location of the practise (Large city vs Medium to small city) | I treat people from Sub-Saharan Africa (No vs Yes) | -- | | 50 | 0.15 | 4.07 | 0.02 | | |
| Family factor  antagonistic behaviour§ | Years of work experience (≤20 years vs >20 years) | I treat people from Europa and North America (No vs Yes) | Employees of the practice can translate (Aways/Often vs Rarely/Never) | Other people can translate (Aways/Often vs Rarely/Never) | | 34 | 0.18 | 1.6 | 0.20 | | |
|  | Years of work experience (≤20 years vs >20 years) | I treat people from Europa and North America (No vs Yes) | Employees of the practice can translate (Aways/Often vs Rarely/Never) | -- | | 48 | 0.14 | 2.46 | 0.07 | | |
|  | Years of work experience (≤20 years vs >20 years) | I treat people from Europa and North America (No vs Yes) | -- | Other people can translate (Aways/Often vs Rarely/Never) | | 35 | 0.11 | 1.26 | 0.30 | | |
|  | Years of work experience (≤20 years vs >20 years) | -- | Employees of the practice can translate (Aways/Often vs Rarely/Never) | Other people can translate (Aways/Often vs Rarely/Never) | | 34 | 0.14 | 1.63 | 0.20 | | |
|  | *--* | *I treat people from Europa and North America (No vs Yes)* | *Employees of the practice can translate (Aways/Often vs Rarely/Never)* | *Other people can translate (Aways/Often vs Rarely/Never)* | | *37* | *0.19* | *2.54* | *0.07* | | |
|  | -- | **I treat people from Europa and North America (No vs Yes)** | **Employees of the practice can translate (Aways/Often vs Rarely/Never)** | **--** | | **51** | **0.14** | **3.81** | **0.03** | | |
|  | -- | I treat people from Europa and North America (No vs Yes) | -- | Other people can translate (Aways/Often vs Rarely/Never) | | 38 | 0.09 | 1.65 | 0.21 | | |
|  | -- | -- | Employees of the practice can translate (Aways/Often vs Rarely/Never) | Other people can translate (Aways/Often vs Rarely/Never) | | 37 | 0.15 | 2.98 | 0.06 | | |
|  | Years of work experience (≤20 years vs >20 years) | I treat people from Europa and North America (No vs Yes) | -- | -- | | 50 | 0.07 | 1.7 | 0.19 | | |
|  | Years of work experience (≤20 years vs >20 years) | -- | Employees of the practice can translate (Aways/Often vs Rarely/Never) | -- | | 48 | 0.12 | 3.01 | 0.06 | | |
|  | -- | **I treat people from Europa and North America (No vs Yes)** | **Employees of the practice can translate (Aways/Often vs Rarely/Never)** | **--** | | **51** | **0.14** | **3.81** | **0.03** | | |
| *§ Due to the large amount of missing values the covariate “Foreign languages spoken – Physicians” was not considered in the development of the final model* | | | | | | | | | | | |
